# Draft Genome Sequence of *Trichomonas tenax* Strain Hs-4:NIH

**DOI:** 10.1128/mra.00157-22

**Published:** 2022-06-16

**Authors:** Nawu Yang, Jevan Christie, Henry L. Keen, Maurice A. Matthew, Chaoqun Yao

**Affiliations:** a Department of Biomedical Sciences, Ross University School of Veterinary Medicine, Basseterre, St. Kitts, West Indies; b One Health Center for Zoonoses and Tropical Veterinary Medicine, Ross University School of Veterinary Medicine, Basseterre, St. Kitts, West Indies; c Iowa Institute of Human Genetics, University of Iowa Carver College of Medicine, University of Iowa, Iowa City, Iowa, United States; University of California, Riverside

## Abstract

*Trichomonas tenax* is a flagellated parasite that plays an important role in periodontal disease, with high prevalence worldwide. Its pathogenesis remains largely unknown, and there is very little information on its genome. Here, we present the whole-genome shotgun sequence of T. tenax strain Hs-4:NIH.

## ANNOUNCEMENT

Trichomonas tenax is usually found colonizing the oral cavity, tartar, and caries of humans and dogs. It is transmitted among hosts by direct contact (kissing), droplet spray, or contaminated food (1). Infection with T. tenax occurs globally with prevalence rates ranging from 4% to 55.9%, depending on geographical location and oral hygiene (2–6). *Trichomonas* protozoa are an ancient eukaryotic lineage without mitochondria; they have a hydrogenosome instead. The latter is a primitive organelle that is phylogenetically related to the fully developed mitochondrion (7, 8). Here, we report a whole-genome shotgun sequence of T. tenax strain Hs-4:NIH, which has great value in medical applications for further studies on the pathogenesis, gene function, and transcriptional regulatory mechanisms of T. tenax.

T. tenax cells (ATCC 30207; ATCC, Manassas, VA, USA) were cultured in Diamond’s medium as described previously (9–12). Briefly, they were incubated at 35°C for 5 days in a round-bottomed Falcon 14-mL tube (Fisher Scientific, Ottawa, Ontario, Canada) filled with 13 mL Diamond’s medium supplemented with 10% horse serum (Sigma-Aldrich, St. Louis, MO, USA) plus 100 U/mL ampicillin and 100 μg/mL streptomycin (Thermo Fisher Scientific, Waltham, MA, USA). Cell density was monitored daily using a hemacytometer (Hausser Scientific, Horsham, PA, USA). Cells at a cell density of 2 × 10^6^ to 5 × 10^6^ cells/mL were collected by centrifugation at 800 × *g* at 4°C for 5 min, followed by three washes in phosphate-buffered saline (pH 7.2) with the same centrifugation. Total genomic DNA was extracted from the pelleted cells using the DNeasy blood and tissue kit (Qiagen, Hilden, Germany). It was shown previously that T. tenax has six haploid chromosomes, each with two parallel chromatids (13). The genome was sequenced 50× in the current study at the Iowa Institute of Human Genetics, University of Iowa, using the NovaSeq 6000 sequencing system (Illumina, San Diego, CA, USA). All programs used in this whole-genome project were used with default settings unless otherwise noted. FastQC (version 0.11.5) was used for quality control assessment of reads. SPAdes (version 3.13.0) was applied for contig construction. Statistics are shown in Table 1. The genome of the closely related trichomonad protozoan Trichomonas vaginalis is highly repetitive, with repetitive sequences accounting for at least 65% of its genome, which was determined by comparing the frequency of observed k-mers to the distribution expected by a Poisson distribution for a repeatless genome (14). The 63.4 Mb of ≥1,000-bp contigs is a little less than one-half of the estimated genome size of 133 Mb for T. tenax, indicating a very repetitive genome like that of T. vaginalis.

**TABLE 1:** Contig statistics without reference^*a*^

Parameter	Finding
No. of contigs	4,908
Largest contig (bp)	212,586
Total length (bp)	63,390,357
*N*_50_ (bp)	38,386
*N*_75_ (bp)	17,150
*L*_50_	469
*L*_75_	1,083
GC content (%)	33.69

a All statistics are based on contigs of ≥1,000-bp size.

The AUGUSTUS program (version 3.4.0) was used for gene prediction using the assembled contigs. A Docker container was created using the Dockerfile available on the AUGUSTUS github site (see below), which was then converted into a Singularity file in order to run on the University of Iowa high-performance computing cluster (https://github.com/Gaius-Augustus/Augustus). The eukaryotic pipeline annotation predicted 4,429 protein-coding genes and 31 small GTPase Rab1 family members.

The InterPro program (versions 5.51 to 85.0) was used for functional prediction. Protein sequences were extracted from the AUGUSTUS output gff files (https://github.com/keenhl/mra_paper). To satisfy the dependency of InterPro on Java (version 11), a special environment was created with Conda. Cysteine proteases are well known virulence factors in T. vaginalis (15). In this draft genome of T. tenax, we were able to identify more than 10 types of proteases, including, but not limited to cysteine protease family C1 related, papain cysteine protease (C1) family, ubiquitin-specific protease and calpain cysteine protease (C2) family, ubiquitin-like-conjugating enzyme ATG3, cysteine proteinase 3, cysteine synthase 1, cathepsin L-like cysteine peptidase, cysteine protease Rdl2 related, and cysteine desulfurylase family, highlighting the diversity of proteases.

In summary, a draft genome sequence of T. tenax was generated in the current study. This genome should lay a foundation for future studies on molecular pathogenesis and host-parasite interactions of this widespread trichomonad protozoan using genomics, proteomics, and transcriptomics. It also makes comparative genomic analyses possible between two highly prevalent human parasites, T. vaginalis and T. tenax.

### Data availability.

Genomic sequences have been deposited in the NCBI Genome Sequence (SUB10957852) and NCBI GenBank (BioProject number PRJNA797354) databases. This whole-genome shotgun project has been deposited at DDBJ/ENA/GenBank under the accession number JALKBU000000000. The version described in this paper is version JALKBU010000000.

## References

[1] Mallat H, Podglajen I, Lavarde V, Mainardi JL, Frappier J, Cornet M. 2004. Molecular characterization of *Trichomonas tenax* causing pulmonary infection. J Clin Microbiol 42:3886–3887. doi:10.1128/JCM.42.8.3886-3887.2004. 15297557 PMC497589

[2] Mehr AK, Zarandi A, Anush K. 2015. Prevalence of oral *Trichomonas tenax* in periodontal lesions of Down syndrome in Tabriz, Iran. J Clin Diagn Res 9:ZC88–ZC90. doi:10.7860/JCDR/2015/14725.6238. PMC4573046 26393213

[3] Eslahi AV, Olfatifar M, Abdoli A, Houshmand E, Johkool MG, Zarabadipour M, Abadi PA, Ghorbani A, Mirzadeh M, Badri M. 2021. The neglected role of *Trichomonas tenax* in oral diseases: a systematic review and meta-analysis. Acta Parasitol 66:715–732. doi:10.1007/s11686-021-00340-4. 33595770

[4] Dimasuay KG, Rivera WL. 2014. First report of *Trichomonas tenax* infections in the Philippines. Parasitol Int 63:400–402. doi:10.1016/j.parint.2013.12.015. 24406842

[5] Dybicz M, Perkowski K, Sędzikowska A, Baltaza W, Chomicz L. 2018. Studies on prevalence of infection with *Trichomonas tenax* identified by molecular techniques – in respect to oral health of patients with various systemic disease requiring immunosuppressive therapy. Ann Parasitol 64:193–197. 30316209

[6] Al Jubory ZHA, Al Hamairy AK. 2021. Molecular study of *Entamoeba gingivalis* and *Trichomonas tenax* among plaque induced gingivitis patients in Babylon province. Ann Rom Soc Cell Biol 25:14012–14027.

[7] Hrdy I, Hirt RP, Dolezal P, Bardonova L, Foster PG, Tachezy J, Embley TM. 2004. *Trichomonas* hydrogenosomes contain the NADH dehydrogenase module of mitochondrial complex I. Nature 432:618–622. doi:10.1038/nature03149. 15577909

[8] Shiflett AM, Johnson PJ. 2010. Mitochondrion-related organelles in eukaryotic protists. Annu Rev Microbiol 64:409–429. doi:10.1146/annurev.micro.62.081307.162826. 20528687 PMC3208401

[9] Diamond LS. 1957. The establishment of various trichomonads of animals and man in axenic cultures. J Parasitol 43:488–490. doi:10.2307/3274682. 13463700

[10] Yao C. 2015. *Tritrichomonas foetus* infections in female beef cattle with abortion in Wyoming, USA. JMM Case Rep 2:e000028. doi:10.1099/jmmcr.0.000028.

[11] Jin Y, Du A, Yao C. 2020. Clinical isolates of *Tritrichomonas foetus* in bulls in Wyoming, South Dakota and Montana, USA. BMC Vet Res 16:12. doi:10.1186/s12917-020-2229-6. 31924216 PMC6954593

[12] Matthew MA, Christie J, Yang N, Yao C. 2022. A loop-mediated isothermal amplification (LAMP) assay specific to *Trichomonas tenax* is suitable for use at point-of-care. Microorganisms 10:594. doi:10.3390/microorganisms10030594. 35336172 PMC8949391

[13] Zubácová Z, Cimbůrek Z, Tachezy J. 2008. Comparative analysis of trichomonad genome sizes and karyotypes. Mol Biochem Parasitol 161:49–54. doi:10.1016/j.molbiopara.2008.06.004. 18606195

[14] Carlton JM, Hirt RP, Silva JC, Delcher AL, Schatz M, Zhao Q, Wortman JR, Bidwell SL, Alsmark UCM, Besteiro S, Sicheritz-Ponten T, Noel CJ, Dacks JB, Foster PG, Simillion C, Van de Peer Y, Miranda-Saavedra D, Barton GJ, Westrop GD, Müller S, Dessi D, Fiori PL, Ren Q, Paulsen I, Zhang H, Bastida-Corcuera FD, Simoes-Barbosa A, Brown MT, Hayes RD, Mukherjee M, Okumura CY, Schneider R, Smith AJ, Vanacova S, Villalvazo M, Haas BJ, Pertea M, Feldblyum TV, Utterback TR, Shu C-L, Osoegawa K, de Jong PJ, Hrdy I, Horvathova L, Zubacova Z, Dolezal P, Malik S-B, Logsdon JM, Henze K, Gupta A, et al. 2007. Draft genome sequence of the sexually transmitted pathogen *Trichomonas vaginalis*. Science 315:207–212. doi:10.1126/science.1132894. 17218520 PMC2080659

[15] León-Félix J, Ortega-López J, Orozco-Solís R, Arroyo R. 2004. Two novel asparaginyl endopeptidase-like cysteine proteinases from the protist *Trichomonas vaginalis*: their evolutionary relationship within the clan CD cysteine proteinases. Gene 335:25–35. doi:10.1016/j.gene.2004.03.002. 15194187

